# Endothelial glycocalyx damage in kidney disease correlates with uraemic toxins and endothelial dysfunction

**DOI:** 10.1186/s12882-020-02219-4

**Published:** 2021-01-10

**Authors:** Hui Liew, Matthew A. Roberts, Alun Pope, Lawrence P. McMahon

**Affiliations:** 1 Department of Renal Medicine, Eastern Health, 3128 Box Hill, Victoria Australia; 2 Eastern Health Clinical School, Monash University, 3128 Box Hill, Victoria Australia

**Keywords:** Glycocalyx, CKD, Uraemia, Sidestream Darkfield, Endothelial dysfunction

## Abstract

**Background:**

Damage to the endothelial glycocalyx is an early indicator of vascular damage and a potential marker of endothelial dysfunction. This study aimed to assess the relationship between markers of glycocalyx damage, endothelial dysfunction, and uraemic toxins in patients with chronic kidney disease.

**Methods:**

Healthy controls, CKD patients, dialysis patients, and kidney transplant recipients had biochemical markers of glycocalyx damage (syndecan-1 and hyaluronan), endothelial dysfunction (von Willebrand factor; vWF and vascular cell adhesion molecule; VCAM-1), and uraemic toxins (indoxyl sulphate and p-cresyl sulphate) measured. In addition, Sidestream Darkfield imaging was performed using the novel GlycoCheck™ device to measure glycocalyx width by the perfused boundary region (PBR) in the sublingual microcirculation.

**Results:**

Serum markers of glycocalyx damage were highest in the dialysis group (*n* = 33), followed by CKD patients (*n* = 32) and kidney transplant recipients (*n* = 30) compared to controls (*n* = 30): hyaluronan: 137 (16-1414), 79 (11–257), 57 (14–218) and 23 (8-116) ng/mL, respectively, *p* < 0.0001; syndecan-1: 81 (40–529), 46 (21–134), 39 (23–72), and 30 (12–138) ng/mL, respectively, *p* < 0.0001. Markers of endothelial dysfunction followed a similar pattern. No difference in the width of the PBR was detected between these groups (2.01 ± 0.35, 2.07 ± 0.27, 2.06 ± 0.28, and 2.05 ± 0.3 µm, respectively, *p* = 0.89). Glycocalyx damage correlated with markers of endothelial dysfunction (log-hyaluronan and log-VCAM-1: *r* = 0.64, *p* < 0.001) and levels of uraemic toxins (log-hyaluronan and log-indoxyl sulphate: *r* = 0.48, *p* < 0.001).

**Conclusions:**

Levels of biochemical markers of glycocalyx and endothelial cell damage are highest in patients receiving dialysis. Glycocalyx and endothelial damage markers correlated with each other, and with uraemic toxins. Although we could not demonstrate a change in PBR, the biochemical markers suggest that glycocalyx damage is most marked in patients with higher levels of uraemic toxins.

## Background

Patients with chronic kidney disease (CKD) have an increased morbidity and mortality from cardiovascular disease. There are many contributory factors, however one postulated pathophysiological mechanism is endothelial dysfunction [1]. The presence of endothelial dysfunction in CKD has been demonstrated by various techniques, including flow-mediated vasodilation and carotid intima-medial thickness, as well as through measurement of serum biomarkers [2–4]. Recent studies have demonstrated the existence of the endothelial glycocalyx, a protective layer overlying the endothelial lining of all blood vessels [5]. It is a potential novel biomarker of early endothelial damage [6]. The glycocalyx is composed of core glycoprotein backbones (e.g. syndecan-1) bound to glycosaminoglycan side-chains (e.g. heparan sulphate and hyaluronan), that interact with adsorbed plasma proteins. Together, these constitute the endothelial surface layer, which has a varying thickness of 0.5 to 8 µm, and which protects the endothelium from damage and governs endothelial permeability to fluid and albumin [7]. Clinical studies have shown that the glycocalyx is damaged in kidney disease, and its damage is closely associated with microalbuminuria and endothelial dysfunction [8–10]. Importantly, studies have demonstrated the potential for therapeutic intervention of this layer [11].

The glycocalyx has been difficult to study as it is easily damaged during vessel handling and techniques used for invasive animal studies are inappropriate for human use. However, elevated serum concentrations of glycocalyx constituents can be measured and correlate with glycocalyx damage [10]. The development of a novel imaging tool (GlycoCheck™) using indirect assessment of the glycocalyx width has enabled non-invasive visualisation of the microcirculation. It has now been used in various clinical diseases including stroke, diabetes, obesity, and kidney disease [12–14].

Damage to the glycocalyx appears to be in proportion with kidney dysfunction. Shed markers are incrementally higher across different CKD stages [10, 13] and removal of the glycocalyx layer in endothelial cell culture models appears to cause increased permeability to albumin [9]. However, while the cause for glycocalyx damage in CKD is not known, exposure to uraemic toxins such as as p-cresyl sulphate (PCS) and indoxyl sulphate (IS) creates endothelial dysfunction [15]. *In vivo* rat studies have also demonstrated increased heparan sulphate shedding in tissues exposed to IS [16]. Therefore, we hypothesized that the glycocalyx is primarily damaged by uraemic toxins, which then directly exposes the endothelial cells to further injury. We aimed to establish the differences in glycocalyx and endothelial dysfunction markers across the different stages of kidney disease and to investigate the associations between markers of the glycocalyx, endothelial dysfunction and uraemic toxins.

## Methods

### Ethics, consent and permissions

This study was approved by the ethics committee at Eastern Health (HREC/15/EH/272, Melbourne, Australia) and written informed consent was obtained from all subjects.

### Patients

We prospectively recruited patients with CKD (eGFR < 60 mL/min/m^2^), dialysis-dependent patients (haemodialysis and peritoneal dialysis), and patients with stable kidney transplants through Eastern Health nephrology clinics and dialysis centres. Patients with known haematological disorders, acute or chronic infections, malignancy and pregnancy were excluded. A control group consisted of healthy volunteers with no known significant or ongoing medical issues, not on medications and with normal kidney function, recruited through advertisement fliers distributed throughout the health service.

### Data collection and blood sampling

Demographic data and clinical information were obtained at enrolment, supplemented by the patients’ medical records when available. Blood and spot urine samples from participants were collected in a fasting state, centrifuged, aliquoted and stored at -80 °C. Biochemical analyses including full blood count, serum creatinine and electrolytes, liver function, c-reactive protein, and urinary albumin:creatinine ratio were performed using standard laboratory techniques (Eastern Health Pathology, Melbourne). Blood samples from haemodialysis patients were obtained pre-dialysis during the midweek session from the arterio-venous fistula or a tunneled dialysis catheter.

### Measurement of glycocalyx and endothelial dysfunction markers

Using enzyme-linked immunosorbent assays, we measured serum hyaluronan (R&D Systems, Minneapolis, MN) and syndecan-1 (Diaclone, Besancon, France) as markers of the glycocalyx, and serum von Willebrand Factor (vWF), (Assaypro, Cambridge, MA) and vascular cell adhesion molecule (VCAM-1), ( R&D Systems, Minneapolis, MN) as markers of endothelial dysfunction. All measures were performed in duplicate.

### Measurement of uraemic toxins

Analyses to quantify indoxyl sulphate (IS) and p-cresyl sulphate (PCS) were performed as previously described by Calaf et al [17], with minor modifications. Plasma samples were deproteinised by adding 300 µl of ethanol to 100 µl of serum, saturated with 100 mg of sodium chloride, followed by 700 µl of mobile phase A and 10 minutes of centrifugation at 10,000 g. Samples were assayed on a Shimadzu ultra-performance liquid chromatography system, with a Merck Lichrospher 60 Select B 5 µ, 125 mm x 4 mm reverse-phase column. Mobile phase A was 20 mM of sodium dihydrogen phosphate and 5 mM tetrabutylammonium iodide in water, and mobile phase B was acetonitrile. All samples were run in duplicates. IS and PCS were quantified on a fluorescence detector at excitation:emission wavelengths of 278:348 nm and 260:285 nm, respectively. Under these conditions, IS and PCS eluted at 7.74 and 11.6 minutes, respectively.

### Sublingual microvessel imaging

Visualisation of the sublingual microvessels was performed using Sidestream Darkfield imaging (Capiscope, KK Technology, Honiton, UK) and the acquired images were analysed using GlycoCheck™ (GlycoCheck BV, Maastricht, Netherlands). In brief, a handheld camera is placed sublingually which, using light-emitting diodes, detects haemoglobin travelling through the microcirculation. The software identifies all measurable microscopic vessels and divides them into 10 µm segments to calculate the transit width of the erythrocyte column. It continues recording until 3000 segments are captured. Measurement of the lateral movement of red blood cells into the glycocalyx region gives the perfused boundary region (PBR). A damaged glycocalyx will allow red blood cells to penetrate further towards the endothelium, thus giving a higher PBR (µm) [18].

### Reproducibility of the PBR in healthy volunteers

To reduce inter-operator bias, all GlycoCheck™ readings were performed by a single operator (HL). To assess intra-operator bias, 6 volunteers had three successive measurements of their PBR taken daily over 4 consecutive days, giving a coefficient of variation of 9.56%.

### Statistics

Nieuwdorp et al. have previously determined that a sample size of 17 was required to detect a 0.2 µm change in glycocalyx width with 80% power and two-sided alpha of 0.05 between groups [19]. Therefore, we aimed to recruit 20 participants in each arm of the cross-sectional study. Data are expressed as absolute values, mean ± standard deviation, or median (range). For normally-distributed data, the Student unpaired t-test was performed for 2-sample comparison and 1-way ANOVA for multiple comparisons. When the data were too non-normal so that nonparametric methods were required, we used Mann-Whitney U test for 2-sample comparisons and Kruskal-Wallis for multiple comparisons. Correlational analyses were performed using Pearson’s correlation coefficient or Kendall’s (when a non-parametric analysis was more appropriate). Because we performed multiple correlations, a Bonferroni-adjusted alpha for the multiple testing was used with a p-value less than 0.05 / 28 = 0.0018 required to be significant.

Univariate and multivariate regression analyses were used to identify predictors of markers of glycocalyx damage. Hyaluronan and syndecan-1 were used as dependent covariates and natural logarithm transformed because of their skewed distribution. Variables assessed for inclusion in the final multivariate regression model included age, gender, diabetes, systolic blood pressure, body mass index (BMI), patient group, eGFR (excluding dialysis patients), p-cresyl sulphate, and indoxyl sulphate.

In each regression model, we fitted large models and then performed stepwise (forward and backward) variable selection with AIC (Akaike’s Information Criterion) as the criterion. The model with minimum AIC was chosen. For simplicity we report only the final model in each case. Data analyses were performed using SPSS (IBM, version 25) and Stata version 15.1 (College Station, Texas) and R version 3.6 (R Foundation for Statistical Computing, Vienna, Austria). A *p*-value of < 0.05 was considered statistically significant, except for the correlation tests, where the significance level was reduced using the conservative Bonferroni correction to 0.05/10 = 0.005, reflecting that there were 10 such tests. Figures were prepared using R.

## Results

### Baseline characteristics

We recruited 30 healthy volunteers, 32 patients with CKD, 33 dialysis patients, and 30 patients with a kidney transplant. We excluded patients who were microcytic, polycythaemic, unable to be venepunctured or unable to have a sublingual assessment performed. Healthy volunteers who were deemed to be hypertensive or overweight at recruitment were also excluded (*n* = 2). Demographic, clinical and biochemical characteristics of the patients and healthy volunteers are outlined in Table 1. Median time on renal replacement therapy was 30 months (1-192) for patients on dialysis, and 20 months (1-164) since transplantation for kidney transplant recipients.

**Table 1:** Baseline clinical and biochemical characteristics of patients

	Dialysis (*n*=33)	CKD (*n*=32)	Transplant (*n*=30)	Healthy controls (*n*=30)	*p*-value
Age (years)	67 (25-82)	71 (37-90)	55 (34-77)	36 (22-69)	<0.0001
Male (%)	26 (79)	24 (75)	17 (57)	11 (37)	0.002
Smoke (%)					
Never	57.6	40.6	60.2	83.3	0.045
Ex-smoker	39.4	53.1	35.8	16.7	
Current smoker	3	6.3	4.1	0	
Diabetes (%)	13 (39.4)	16 (50)	11 (36.7)	0	0.527*
IHD (%)	9 (27)	8 (25)	7 (23)	0	0.937*
Hypertension (%)	24 (73)	26 (81)	22 (73)	0	0.675*
PVD (%)	2 (6)	1 (3)	3 (10)	0	0.537*
CVA (%)	1 (3)	2 (6)	2 (7)	0	0.775*
BMI (kg/m^2^)	29 ± 5	30 ± 9	26 ± 4	24 ± 3	0.001
SBP (mmHg)	141 ± 26	144 ± 21	142 ± 16	118 ± 10	<0.0001
Hb (g/L)	120 ± 11	129 ± 15	133 ± 18	140 ± 12	<0.0001
MCV (fL)	89 ± 7	88 ± 4	90 ± 6	87 ± 3	0.167
eGFR (mL/min/1.73m^2^)	-	31 (10-59)	59 (32-90)	98 (70-127)	<0.0001
Urea (mmol/L)	18.7 ± 6.2	13.5 ± 4.8	9.1 ± 2.5	4.7 ± 1.1	<0.0001
ALT (IU/L)	16 ± 8	24 ± 22	23 ± 11	22 ± 11	0.100
GGT(IU/L)	23 (10-259)	37 (8-163)	24 (9-233)	17 (10-40)	0.028
Albumin (g/L)	34 ± 4	38 ± 4	39 ± 3	41 ± 2	<0.0001
Glucose (mmol/L)	6.4 (2-13)	5.6 (4.5-18)	4.9 (3-19)	4.8 (4.2-5.8)	<0.0001
CRP (mg/L)	4 (0-38)	6 (2-29)	4 (2-147)	0 (0-11)	<0.0001
p-Cresyl sulphate (μM)	52 ± 33	29 ± 20	9 ± 11	6 ± 4	<0.0001
Indoxyl sulphate (μM)	19.2 ± 12.1	3.8 ± 4.6	2.2 ± 2.7	1.1 ± 0.4	<0.0001

Data are presented as mean ± standard deviation or median (range). eGFR values are calculated using the CKD-EPI formula.

*Chi-square analyses performed between CKD, Dialysis and Transplant groups only.

*IHD* ischaemic heart disease, *PVD* peripheral vascular disease, *CVA* cerebrovascular accident, *BMI* body mass index, *SBP* systolic blood pressure, *DBP* diastolic blood pressure, *MCV* mean corpuscular volume, *ALT* alanine transferase, *GGT* gamma glutamyl transferase

### Serum markers of the glycocalyx and endothelial dysfunction worsen with CKD progression

Hyaluronan and syndecan-1 levels were highest in the dialysis group, followed by the CKD and transplant groups compared to controls (*p* < 0.0001). vWF and VCAM-1 levels were similarly elevated, *p* < 0.0001 (Table 2).

**Table 2:** Glycocalyx and endothelial dysfunction markers of patients according to selected group

	Healthy controls *n* = 30	Transplant *n* = 30	CKD *n* = 32	Dialysis *n* = 33	*p*-value
PBR (µm)	2.05 ± 0.3	2.06 ± 0.28	2.07 ± 0.27	2.01 ± 0.35	0.89
Hyaluronan (ng/mL)	23 (8-116)	57 (14–218)	79 (11–257)	137 (16-1414)	< 0.001
Syndecan-1 (ng/mL)	30 (12–138)	39 (23–72)	46 (21–134)	81 (40–529)	< 0.001
vWF (mIU/mL)	1685 ± 689	2016 ± 653	3076 ± 946	3920 ± 1755	< 0.001
VCAM-1 (ng/mL)	598 (345–1189)	927 (602–1813)	1176 (559–3046)	1479 (737–2900)	< 0.001
uACR (mg/mmol)	0.4 (0-1.9)	5 (0–35)	30 (0-730)	-	0.002

### Serum markers of the glycocalyx correlate with markers of endothelial dysfunction

**Fig. 1:**
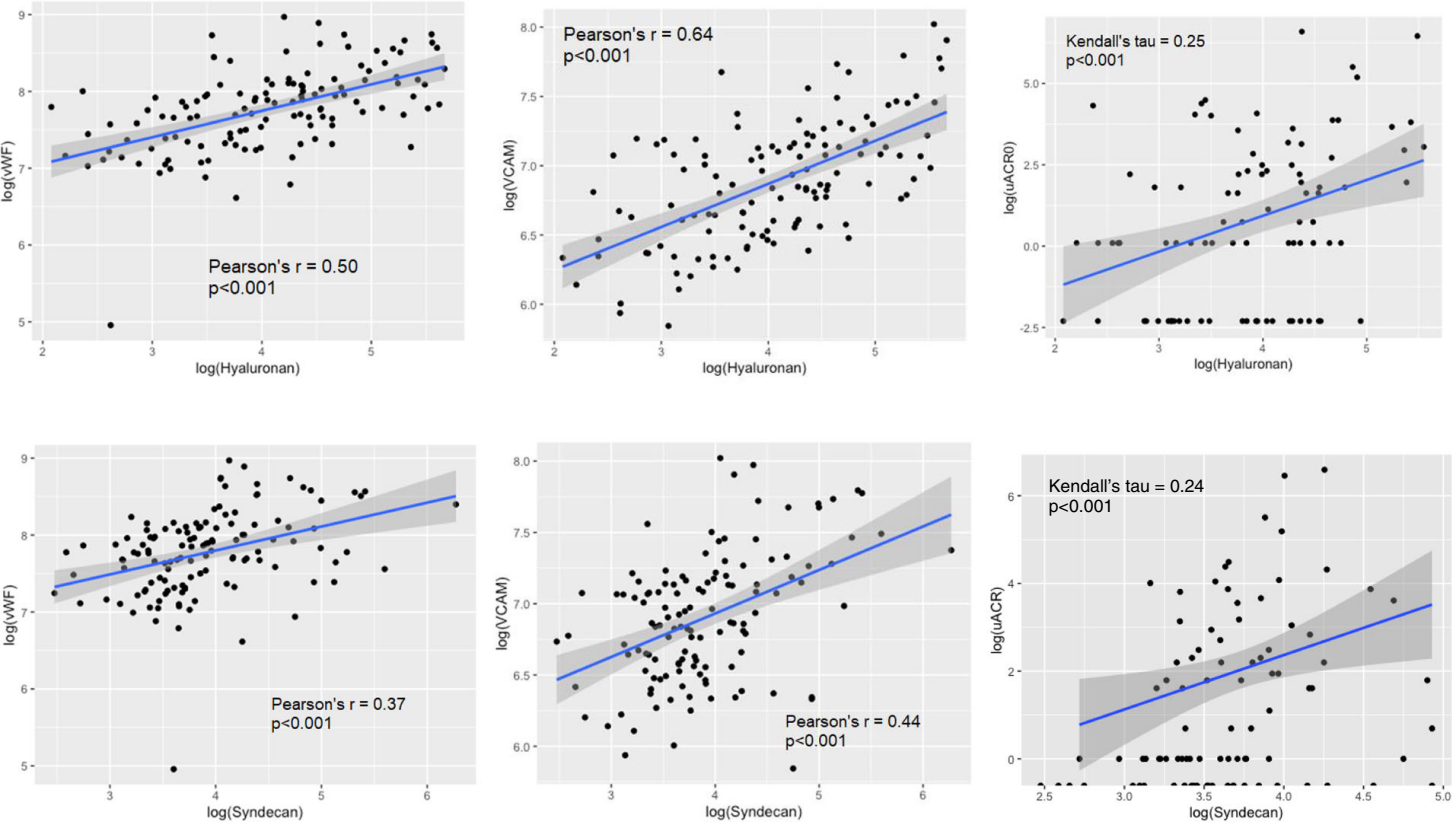
Correlation graphs of markers of glycocalyx damage and endothelial dysfunction. Both log-hyaluronan and log-syndecan-1 correlated with log-VCAM-1, log-vWF, and log-uACR

To counteract skewness, all concentrations were log-transformed. Log-hyaluronan and log-syndecan-1 levels correlated with each other (*r* = 0.37, *p* < 0.001), and log-VCAM-1 and log-vWF correlated with each other (*r* = 0.56, *p* < 0.0001). Log-hyaluronan correlated with log-VCAM-1 (*r* = 0.64, *p* < 0.0001), log-vWF (*r* = 0.50, *p* < 0.0001), and log-uACR (Kendall’s tau = 0.25, *p* < 0.001). Log-syndecan-1 correlated with log-VCAM-1 (*r* = 0.44, *p* < 0.0001), log-vWF (0.37, *p* < 0.001), and log-uACR (Kendall’s tau = 0.24, *p* < 0.001). (Fig. 1).

### Serum markers of the glycocalyx correlate with uraemic toxins

**Fig. 2:**
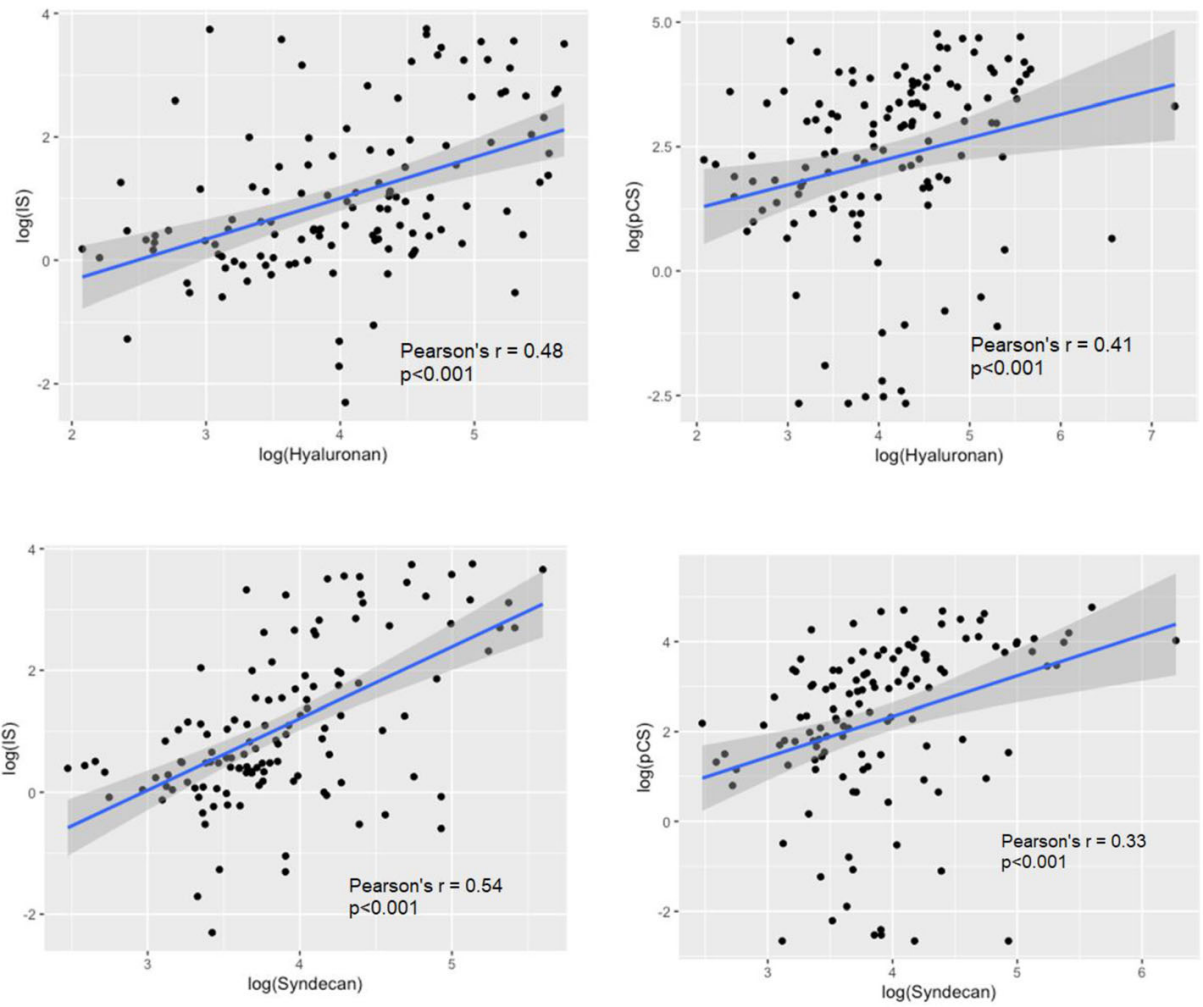
Correlation graphs of glycocalyx markers and uraemic toxins. Both log-hyaluronan and log-syndecan-1 correlated with log-indoxyl sulphate and log-p-cresyl sulphate

Log-IS and log-PCS correlated with each other (*r* = 0.55, *p* < 0.0001), and with glycocalyx markers (log-IS and log-hyaluronan, *r* = 0.45, *p* < 0.0001; log-IS and log-syndecan-1, *r* = 0.58, *p* < 0.001; log-PCS and log-hyaluronan, *r* = 0.41, *p* < 0.001; log-PCS and log-syndecan-1, *r* = 0.32, *p* < 0.001) (Fig. 2). Log-IS and log-PCS also correlated with markers of endothelial dysfunction (log-IS and log-VCAM-1, *r* = 0.55, *p* < 0.0001; IS and log-vWF, *r* = 0.30, *p* = 0.0005; log-PCS and log-VCAM-1, *r* = 0.52, *p* < 0.0001; log-PCS and log-vWF, *r* = 0.40, *p* < 0.0001). The p-values for testing each coefficient are all < 0.001, so all the correlations are significantly different from 0 after Bonferroni correction.

### Predictors of serum markers of the glycocalyx

#### Hyaluronan

Univariate analysis indicated that age, eGFR (excluding dialysis patients), SBP, diabetes, BMI, patient group, log-IS and log-PCS were associated with log-hyaluronan. Age, patient group, and log-IS demonstrated the strongest influence based on the highest R^2^ values (Table 3). Patient group was included first as the strongest predictor and the R^2^ value increased with addition separately of SBP (R^2^ = 0.41, LR *p* < 0.002) and age (R^2^ = 0.50, LR *p* < 0.001). When SBP was added to the model including patient group and age, it did not explain extra variance (LR *p* = 0.09). Although the association of log-IS was strong on its own, this was attenuated when adjusted for patient group (beta coefficient reduced to 0.12, *p* = 0.17) and therefore included in the final model, which determined that only age and group were significantly associated with log-hyaluronan, R^2^ = 0.50 (Table 3). Addition of the other variables did not explain additional variance in the log-hyaluronan at the *p* < 0.05 level. Introducing interactions and performing stepwise model selection led to the same reduced model, containing no significant interactions.

**Table 3:** Regression analyses of log-hyaluronan

Variables	Univariable coefficient β (95% CI)	*p*-value	R^2^	Multivariable coefficient β (95% CI)	*p*-value
Age	0.03 (0.02 to 0.04)	<0.001	0.41	0.028 (0.02 to 0.04)	<0.001
eGFR^b^	-0.01 (-0.01 to -0.003)	0.003	0.09		
SBP	0.02 (0.02 to 0.03)	<0.001	0.19		
BMI	0.03 (-0.009 to 0.06)	0.01	0.05		
Patient group^a^			0.36		
CKD	1.13 (0.75 to 1.50)	<0.001		0.30 (-0.14 to 0.73)	0.18
Dialysis	1.52 (1.15 to 1.90)	<0.001		0.87 (0.46 to 1.26)	<0.001
Transplant	0.77 (0.38 to 1.15)	<0.001		0.33 (-0.04 to 0.70)	0.08
Diabetes	0.35 (0.004 to 0.70)	0.05	0.03		
Log-IS	0.35 (0.24 to 0.47)	<0.001	0.23		
Log-PCS	0.12 (0.03 to 0.21)	0.01	0.06		

Multivariable analysis model R^2^=0.50. However, when we tested for interactions between age and group, there were no significant interactions. The estimates for group changed significantly but not for age

^a^Compared to healthy controls as the reference group

^b^Excludes dialysis values

#### Syndecan-1

For log-syndecan-1, age, gender, patient group, eGFR, SBP, diabetes, log-IS and log-PCS demonstrated significant associations (Table 4), with ‘patient group’ having the greatest influence (R^2^ = 0.41). Inclusion of log-IS in the model with ‘patient group’ attenuated the association of log-IS with log-syndecan-1 (adjusted beta-coefficient 0.05, *p* = 0.41). Therefore, the best model for syndecan-1 included ‘patient group’ only (Table 4). Addition of the other variables did not explain additional variance in log-syndecan-1 at the *p* < 0.05 level.

**Table 4:** Regression analyses of log-syndecan-1

Variables	Univariable coefficient β (95% CI)	*p*-value	R^2^	Multivariable coefficient β (95% CI)	*p*-value
Age	0.008 (0.0006 to 0.015)	0.03	0.04		
Gender	0.40 (0.17 to 0.63)	0.001	0.09		
eGFR^b^	-0.004 (-0.007 to -0.0004)	0.03	0.05		
SBP	0.007 (0.001 to 0.01)	0.01	0.05		
Diabetes	0.29 (0.04 to 0.54)	0.02	0.04		
Patient group^a^			0.41		
CKD	0.36 (0.10 to 0.62)	0.01		0.36 (0.10 to 0.62)	0.006
Dialysis	1.10 (0.84 to 1.35)	<0.001		1.10 (0.84 to 1.35)	<0.001
Transplant	0.18 (-0.08 to 0.44)	0.18		0.18 (-0.08 to 0.44)	0.181
Log-IS	0.28 (0.20 to 0.36)	<0.001	0.30		
Log-PCS	0.12 (0.06 to 0.18)	<0.001	0.11		

Multivariable analysis model R^2^=0.41

^a^Compared to healthy controls as the reference group

^b^Excludes dialysis values

### Association with PBR

No changes between groups were detected in the PBR (Table 2). PBR also did not correlate with serum markers of glycocalyx or endothelial dysfunction.

## Discussion

There has been growing interest in the endothelial glycocalyx in recent decades, with increasing appreciation of its significance in vascular health. Given the importance of vascular regulation and endothelial function in kidney disease, glycocalyx research in this field has also flourished recently. With 125 participants, to the best of our knowledge, this study is the largest to incorporate both imaging and biochemical markers of the glycocalyx. Additionally, it is the first study of the glycocalyx to report data on uraemic toxin concentrations, correlating IS and PCS levels with markers of the glycocalyx. Consistent with other reports, it showed increasing serum levels of the glycocalyx at different stages of kidney disease. Whilst the exact cause for this remains uncertain, the correlation between increased IS and PCS levels with increased serum markers of glycocalyx injury suggests that uraemic toxins play a role in the degradation of this layer. Uraemic patients have a high incidence of vascular disease, and uraemic toxins play an important role in the progression of cardiovascular disease in patients with kidney disease. IS and PCS are two commonly-studied toxins and both have been associated with vascular disease and mortality in this population [20]. IS induces oxidative stress in endothelial cells, stimulates vascular smooth muscle cell proliferation, and promotes aortic calcification; [21, 22] whereas PCS induces endothelial microparticle release and causes apoptosis in cardiac myocytes [23, 24]. Furthermore, IS and PCS have been shown to promote the interaction between leukocytes and the endothelium, possibly due to degradation of the protective glycocalyx layer [16]. Other potential mechanisms contributing to glycocalyx damage in CKD include hypervolaemic states, sodium excess, and the generation of inflammatory cytokines and reactive oxygen species [25–27].

While this study is not powered to assess survival outcomes, a high serum hyaluronan concentration has been associated with poor survival in patients with kidney disease [28] and syndecan-1 has been shown to be elevated in patients with acute coronary syndrome and decompensated heart failure [29]. In our study, both hyaluronan and syndecan-1 were correlated with the degree of microalbuminuria. The median syndecan-1 level of our control subjects was comparable with other studies [30, 31] although the results have varied from 17 to 50 ng/mL in healthy controls of other studies [13, 32]. The median hyaluronan concentration of our control subjects was lower than that reported in some studies [10, 33] but other research similarly reports a wide range from 17 to 86 ng/mL, and our control hyaluronan levels are comparable to another study involving dialysis patients [30, 33].

Assessment of the glycocalyx using *in vivo* imaging is an indirect method of estimating its thickness. Sidestream Darkfield imaging is a non-invasive method of assessing the microcirculation, and is used where tissues are trans-illuminable (such as the eyes, lungs and even kidneys) [34–36]. Studies have mainly focused on the sublingual microcirculation as it is the most readily-accessible mucosal surface of the human body. Sublingual vessels arise from the external carotid artery and have been used as a surrogate of the splanchnic circulation given the shared embryogenic origin of the tongue with the gut [37]. However, whether one can extrapolate the changes in one microcirculatory bed to another is debatable. Direct placement of the SDF probe on kidney to compare the microvascular parameters between the sublingual and renal cortical microcirculation in a porcine study did not show a correlation [38]. Other studies reported a correlation between sublingual and intestinal microcirculatory alterations only when the disease became more systemic, such as with generalised sepsis [39].

Previously, estimating the glycocalyx with *in vivo* studies involved manual calculations of the difference between a vessel’s width before and after the passage of a leukocyte at a single reference point. This process is time-consuming and subject to bias. The GlycoCheck™ software bypasses this by performing fully-automated calculations and estimates glycocalyx width based on vessel perfusion and erythrocyte movement. As previously mentioned, this tool has been used in the research of different clinical scenarios. However, unlike other studies, we were unable to detect a difference in the PBR between the different groups of our study. First, this may be due to the cohort of patients recruited in this study. Positive studies using the GlycoCheck™ have assessed the very ill—such as those with sepsis, admitted to the intensive care unit, or undergoing cardiac bypass surgery; [40, 41] whereas the patients recruited in this study were all stable outpatients with no acute medical illness.

Second, there were only a small number of participants recruited. Sample size calculations based on other studies using the same statistical power and significance also suggest a sample size of 12 to 26 per group [12, 13, 42]. Despite recruiting almost twice the recommended number of participants, 30 per group may still be inadequate to detect glycocalyx changes by PBR alone. Furthermore, there is currently no defined range of normal PBR values, and the available literature reports a wide variation from 1.79 to 3.3 µm in healthy controls [30, 43]. Given the wide range of possible ‘normal’ readings, it is difficult to determine what degree of glycocalyx width reduction is considered significant, although 0.2 µm is currently taken to be the target [19]. The standard deviation of our PBR readings was slightly greater than other studies, although our coefficient of variance of 9.56% should indicate good PBR reproducibility. A reproducibility study of the GlycoCheck™ demonstrated a good intraclass correlation between observers, but also demonstrated a wide range of variability in the readings [44]. The inherent operator-dependency (with variable focus, pressure and stability considerations) was nullified as far as possible by using a single operator who demonstrated acceptable variance in repeated measures. Nonetheless, our study did not support using the PBR as the sole determinant of glycocalyx structure. Whether PBR assessments over time are more useful than a single reading is uncertain. In our study, there was a large variability in PBR readings within each group, which may account for the limited difference between them. Individual changes in PBR assessments may well clarify changes when patients are used as their own controls over time.

There are other potential confounders of the PBR that have not yet been investigated. For example, the effect of vasodilation and vasoconstriction on the calculation of the PBR has not been clarified. This is important in the context of endothelial dysfunction where there is impaired nitric oxide availability and defective vasodilation. Another consideration is the unknown impact of erythrocyte shape, size and quantity on how the PBR is calculated. Participants with known haematological disorders were excluded from this study as PBR measurement may be affected, though this is unproven. Furthermore, the effect of food intake on the glycocalyx and PBR has not been investigated. Blood samples in this study were obtained in a fasting state, as did one study [30], but not another [13], and PBR measurements were not repeated after food. Caffeine has been shown to affect vessel perfusion, but not the PBR [45].

We elected to use syndecan-1 and hyaluronan as markers of the glycocalyx as they represent different structural components of the glycocalyx. However, they are not specific to the glycocalyx. Hyaluronan is present on connective and skin tissues, [46] whereas syndecan-1 is also expressed on epithelial cells, plasma cells, and hepatocytes [47]. Therefore, detection of these components in the bloodstream is not specific to the injury of any particular organ. Additionally, the exact physiology and metabolism of the glycocalyx is not well-defined. It is thought to be in a constant state of synthesis and damage, but the impact of renal impairment on these glycocalyx markers needs further exploration. Hyaluronan has a high molecular weight of up to 4000 kDa and is degraded by hyaluronidases into smaller fragments [48]. Hyaluronan is predominantly cleared by the liver, with a small component being cleared in the urine [49]. Given the normal hepatic function of our participants, measured hyaluronan probably originated from the endothelium or elsewhere, rather than concentrations being elevated due to reduced hepatic clearance. On the other hand, the clearance of syndecan-1 has thus far not been reported. The molecular mass of syndecan-1 is variable due to variation in the length of heparan sulfate chains attached to it, and therefore can range from 32 to 200 kDa. Regardless, the possible contribution of reduced renal excretion in the interpretation of elevated glycocalyx concentrations is acknowledged. In our study, serum hyaluronan negatively correlated with eGFR (*r*=-0.47, *p* < 0.001), whereas serum syndecan-1 did not (*r*=-0.117, *p* = 0.25). In univariate regression analyses, eGFR was associated with both log-hyaluronan and log-syndecan-1 and ‘patient group’ was a significant predictor in multivariable regression, suggesting reduced excretion when renal function is impaired. Of note, both hyaluronan and syndecan-1 are measurable in the urine in health and in disease, [50, 51] but the significance of urine concentrations of both molecules in the context of kidney disease is unclear.

Our CKD cohort was significantly older than the other participants in this study. Relatively little has been published about how the glycocalyx is affected by age in humans. One study has shown a greater PBR in older humans, [52] whereas a large multiethnic population study did not show any effects of age on the PBR [53]. Another study on skeletal muscle ageing did not find a significant difference between younger and older healthy men, unless they had diabetes [43]. Thus, the effects of age on the glycocalyx are conflicting, which suggests that age alone may not be the sole cause of glycocalyx damage, but rather the accumulation of vascular comorbidities over time. In this study, the prevalence of macrovascular disease was low and unlikely to be a confounding factor.

## Conclusions

This study demonstrates an association between the biochemical markers of glycocalyx damage and the presence of uraemic toxins in patients with CKD. Future studies to determine the metabolism of the glycocalyx, how its serum markers are affected by renal clearance and direct *in vivo* or *in vitro* effects of uraemic toxins on the glycocalyx will increase our understanding of the role of the glycocalyx in protecting the cardiovascular health of patients with CKD, thus potentially paving a way for novel therapeutic strategies.

## Data Availability

All data generated or analysed during this study are included in this published article (and its supplementary information files).
